# Short- and Long-Term Outcome of 71 Foals Undergoing Omphalectomy with Peritonealization of Arterial Stumps

**DOI:** 10.3390/ani16040551

**Published:** 2026-02-10

**Authors:** Maria Virginia Ralletti, Federica Meistro, Riccardo Rinnovati, Paola D’Angelo, Jole Mariella, Alessandro Spadari

**Affiliations:** Department of Veterinary Medical Sciences, University of Bologna, Ozzano dell’Emilia, 40064 Bologna, Italy; federica.meistro@unibo.it (F.M.); riccardo.rinnovati2@unibo.it (R.R.); paola.dangelo7@unibo.it (P.D.); jole.mariella2@unibo.it (J.M.); alessandro.spadari@unibo.it (A.S.)

**Keywords:** foal, omphalectomy, neonatal surgery, peritonealization, abdominal adhesions, colic, long-term outcome

## Abstract

Umbilical remnant disorders are common in neonatal foals and may require surgical intervention when complicated by infection or urinary involvement. Abdominal surgery in this population is often considered high risk, particularly due to postoperative complications and adhesion formation. The aim of this retrospective observational study was to evaluate the short- and long-term outcomes of foals undergoing omphalectomy with peritonealization of the umbilical arterial stumps. Medical records of foals younger than 30 days treated surgically between 2005 and 2025 were reviewed. Data regarding signalment, diagnosis, comorbidities, postoperative complications, and short-term survival were collected. Long-term follow-up was obtained by owner interview to assess post-discharge colic episodes and if they started their intended athletic activity. Seventy-one foals met the inclusion criteria. Overall, 83.1% survived to hospital discharge. Mortality was significantly associated with the presence of concurrent comorbidities and postoperative complications, but not with age or sex. Long-term follow-up was available for a subset of foals; post-discharge colic was reported in 6.4% of cases, with only one foal developing colic considered compatible with postoperative adhesions. Most foals with available follow-up successfully started their expected athletic activity. These findings suggest that omphalectomy with peritonealization of the umbilical arterial stumps is associated with favorable short- and long-term outcomes and may represent a safe surgical option in neonatal foals.

## Simple Summary

Umbilical disorders are common in neonatal foals and may involve infection of the umbilical vessels or urachal structures, potentially leading to severe systemic complications. Although medical management is often attempted initially, surgical intervention is frequently required in complicated cases. Abdominal surgery in neonatal foals is traditionally considered high risk, particularly because of concerns regarding postoperative infection and intra-abdominal adhesion formation, which may result in colic later in life. This study evaluated the short- and long-term outcomes of 71 neonatal foals that underwent surgical removal of umbilical structures using a technique that included peritonealization of the umbilical arterial stumps. Most foals survived to hospital discharge, and deaths were mainly related to severe concurrent illnesses rather than the surgery itself. Long-term follow-up showed that only a small number of foals developed colic after surgery, and only one case was diagnosed with abdominal adhesions. Most foals for which sport activity follow-up was available started their expected athletic activity. These results suggest that this surgical technique is safe and may help reduce long-term abdominal complications associated with adhesions, improving both animal welfare and future performance.

## 1. Introduction

Umbilical disorders are among the most common conditions affecting neonatal foals, potentially involving any of the anatomical remnants of the fetal umbilical cord, including the umbilical arteries, umbilical vein, and urachus. These structures undergo a complex process of postnatal involution, and any disruption of this process can predispose the neonate to localized or systemic disease. Among the most frequently reported conditions are omphalitis, omphalophlebitis, omphaloarteritis, patent urachus, urachal rupture, and umbilical hernia, each associated with variable degrees of morbidity and, in severe cases, life-threatening complications [1,2,3]. Although initial management traditionally relies on medical treatment, definitive resolution of complicated cases often requires surgical intervention. The surgical approach to umbilical remnants has shown high efficacy with survival rates ranging from 66 to 91% [1,4,5,6,7]. The surgical technique for remnant resection is thoroughly documented [1], and variations such as laparoscopic-assisted resection [8], the two-step omphalectomy technique using the LigaSure™ (LigaSure Atlas™, Covidien Medtronic, Minneapolis, MN, USA) [9] and marsupialization of the umbilical vein [4,10,11,12] have been successfully reported. Postoperative complications such as peritonitis, wound dehiscence, and adhesion formation remain possible, particularly in debilitated foals.

Despite the widespread perception that abdominal surgery in neonatal foals carries a high risk of complications, evidence suggests that, when performed promptly and using appropriate surgical methods, it can be a safe and effective therapeutic option [2,5].

Adhesion formation is a main complication of abdominal surgery with a prevalence of up to 33% in adult equine patients [13] and in foals [14] and is recognized as among the most common causes of colic syndrome in horses undergoing repeat celiotomy after previous abdominal surgery [15]. Inflammation and the presence of infected tissue are among the possible trigger mechanisms of adhesion development [16]. The peritoneum can play an important role in preventing adhesions over umbilical vascular stumps because it regulates inflammatory responses and prevents fibrosis in the abdominal cavity [17].

However, despite the broad use of various surgical techniques, no studies have specifically evaluated the short- and long-term outcome of omphalectomy performed in association with peritonealization of the umbilical arterial stumps in foals. This technical variation, based on covering the transected arterial stumps with a visceral-vesical peritoneal flap, is hypothesized to reduce the risk of postoperative adhesions by minimizing serosal exposure, isolating potentially contaminated tissues, and enhancing peritoneal healing. To date, its efficacy in preventing adhesion formation has not yet been systematically documented in the veterinary literature.

In this context, the aim of the present study was expanded to evaluate not only survival but also clinically relevant long-term complications, including colic episodes possibly associated with postoperative adhesions in foals undergoing omphalectomy with peritonealization of the umbilical arterial stumps.

Short- and long-term outcomes, including the start of their intended activity and occurrence of colic episodes potentially related to adhesions, were assessed to provide novel evidence on the potential protective role of this surgical technique.

## 2. Materials and Methods

### 2.1. Study Design and Case Selection

This retrospective observational study reviewed medical and surgical records of neonatal foals admitted to the Veterinary Teaching Hospital between June 2005 and July 2025 for umbilical remnant disease requiring surgery. Only foals younger than 30 days at the time of admission were included, as this age window encompasses the period in which umbilical structures have not yet undergone complete physiological involution and clinical diseases of the umbilical remnants most commonly manifest. Foals were selected if they had been diagnosed with omphalitis, omphaloarteritis, omphalophlebitis, patent urachus, urachal rupture, bladder rupture, or combinations thereof, and if surgical treatment involved resection of the umbilical remnants performed with a standardized technique incorporating peritonealization of the umbilical arterial stumps. Foals were excluded if they were ≥30 days old, if medical documentation was incomplete, or if surgical management deviated from the technique described.

### 2.2. Data Collection

Data were collected retrospectively from the electronic medical records and surgical reports of all foals meeting the inclusion criteria. For each case, information regarding age at admission, sex, breed, and year of presentation was retrieved from the clinical database. The admitting diagnosis and the specific umbilical structures involved, namely the umbilical vein, one or both umbilical arteries, and the urachus, were reported. When documented, concurrent comorbidities such as failure of passive transfer, sepsis, neonatal maladjustment syndrome, septic arthritis, flexural or angular limb deformities, pneumonia, and diarrhea were also incorporated into the dataset.

Information regarding the postoperative course was extracted from hospitalization notes and included any complication recorded after surgery, with particular attention to those directly related to the surgical procedure, such as incisional complications, postoperative pyrexia, or colic episodes documented during recovery. Duration of hospitalization and short-term outcome, defined as survival to hospital discharge or death/euthanasia before discharge, were also obtained directly from the medical records.

Long-term follow-up was performed by telephone interview with the owners of all surviving foals. Owners were asked whether the horse had experienced any episode of colic after discharge (yes/no) and, if so, to describe the nature of the episode. No additional diagnostic investigations were performed to confirm the presence of postoperative adhesions; the classification of colic episodes therefore reflected exclusively the history reported by owners. Owners were also asked whether the horse had started the athletic activity expected for its breed and intended use. When available, this information was supplemented by searching publicly accessible online registries and performance databases to confirm participation in training or competition. Because the objective was to determine whether the horse had started its intended activity rather than to quantify performance level, athletic outcome was recorded simply as starting the expected activity (yes/no). Only horses for which follow-up information could be obtained were included in the long-term outcome analysis.

### 2.3. Surgical Procedure

**Figure 1:**
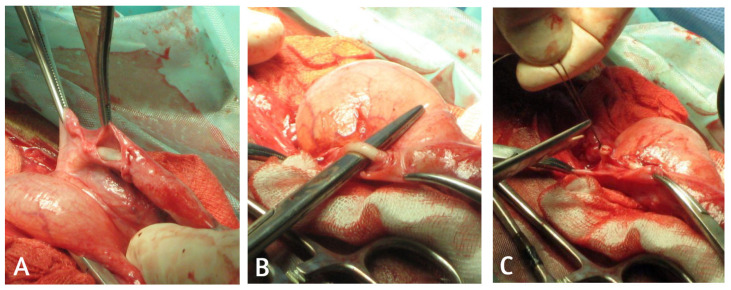
(**A**) Dissection of the peritoneal portion of the bladder’s lateral ligament surrounding the vascular adventitial layer. (**B**) Identification and isolation of the umbilical artery. (**C**) Following single ligation with 2/0 USP Biosyn, the artery is resected and the surrounding peritoneal flap is used to cover the arterial stump using a short continuous inverting suture with 2/0 USP Biosyn.

All surgeries were performed under inhalant general anesthesia with the foal positioned in dorsal recumbency. After standard aseptic preparation, a sharp elliptical skin incision was made around the umbilicus, extended cranially and caudally to expose the umbilical structures. Once isolated, the umbilical vein was double ligated with Biosyn 2/0 USP (Medtronic, Minneapolis, MN, USA) and resected. The umbilical stump and the apex of the bladder were then exteriorized caudally to enable visualization of the umbilical arteries and urachus. A peritoneal flap was created by dissecting the peritoneal portion of the bladder’s lateral ligament surrounding the vascular adventitial layer (Figure 1); subsequently a single ligation using Biosyn 2/0 USP was done on each artery. Following artery resection, the peritoneal flaps were mobilized to cover each arterial stump and secured with a short continuous inverting suture using 2/0 USP Biosyn in order to minimize exposure of suture material and consequently reduce serosal irritation, promoting rapid healing of the mesothelium and a lower inflammatory response.

Subsequently, the urachus was resected following the placement of an enterostat clamp at the apex of the bladder. Cystoplasty was performed through two layers of Cushing sutures with Biosyn 2/0 USP, ensuring that the deeper layer did not incorporate the submucosa. Before repositioning the bladder into the abdomen, a suture integrity test was performed by instilling sterile saline through the urinary catheter. The abdomen closure was realized in three layers. This surgical technique, including peritonealization of the arterial stumps, was applied consistently across all cases included in the study.

### 2.4. Post-Operative Management

Post-operatively, all foals were administered antimicrobial therapy for one week based on blood or umbilical discharge culture. Flunixin meglumine 1.1 mg/kg IV BID was administered for 3 to 5 days. Sucralfate 20 mg/kg orally QID was given as gastroprotection. Foals were also managed with urinary catheterization, protection of the surgical wound using a foam bandage during the first hours, and stall confinement; abdominal bandaging was not routinely applied and was required only in selected cases. Foals with comorbidities received additional and specific therapy for each identified pathology.

### 2.5. Statistical Analysis

Normality of continuous variables (age at admission and duration of hospitalization) was evaluated using the Shapiro–Wilk test. Continuous variables were compared between outcome groups using the Mann–Whitney U test, as these data were not normally distributed. Categorical variables, including comorbidities, postoperative complications, and short-term and long-term outcomes, were compared between groups using the chi-square test or Fisher’s exact test. Statistical significance was set at *p* < 0.05. All statistical analyses were performed using IBM SPSS Statistics for Windows, Version 29.0 (IBM Corp., Armonk, NY, USA).

## 3. Results

### 3.1. Study Population

A total of 71 neonatal foals met the inclusion criteria and were included in the study. The population consisted predominantly of male foals (50/71; 70.4%), whereas 21/71 foals (29.6%) were females. The median age at admission was 5 days (interquartile range [IQR] 3–9 days). Umbilical disorders included conditions such as omphalitis, urachal disorders, and uroperitoneum secondary to bladder rupture, as well as mixed presentations. Concurrent comorbidities were commonly recorded, and at least one comorbidity was documented in 38/71 foals (53.5%). Failure of passive transfer was identified in 14/71 foals (19.7%). Septic conditions were documented in 11/71 foals (15.5%), including septicemia (5), septic arthritis (8), and osteomyelitis (2); some foals presented with more than one septic manifestation. Additional comorbidities included limb deformities (9), prematurity (4), cryptosporidiosis (3), diarrhea/enteritis (1), and other concurrent conditions (16).

### 3.2. Short-Term Outcome

Overall, 59 foals (83.1%) survived to hospital discharge, whereas 12 foals (16.9%) did not survive to discharge due to death or euthanasia during hospitalization. Two additional deaths were reported after hospital discharge during the follow-up period. The median duration of hospitalization for the entire cohort was 13 days (IQR 9–20 days). Normality testing using the Shapiro–Wilk test demonstrated that both age at admission and duration of hospitalization were not normally distributed (*p* < 0.05). Consequently, non-parametric tests were used for comparisons between outcome groups. Foals that did not survive to discharge had a significantly shorter hospitalization period compared with survivors (median 8 vs. 15 days, respectively; Mann–Whitney U test, *p* = 0.011). In contrast, age at admission did not differ significantly between foals that survived to discharge and those that did not (median 6 vs. 4 days, respectively; Mann–Whitney U test, *p* = 0.233).

### 3.3. Postoperative Complications

No intra-operative complications were recorded in any of the included cases. Postoperative complications were documented in 9/71 foals (12.7%) and included incisional complications (7/9), postoperative pyrexia (2/9), and systemic deterioration requiring additional medical treatment (1/9). The occurrence of postoperative complications was significantly associated with a negative short-term outcome. Postoperative complications were observed in 4/12 foals (33.3%) that did not survive to discharge, compared with 5/59 foals (8.5%) that survived (Fisher’s exact test, *p* = 0.039). Foals that did not survive to discharge were significantly more likely to present with one or more concurrent comorbidities at admission compared with survivors (10/12; 83.3% vs. 28/59; 47.5%, respectively; Fisher’s exact test, *p* = 0.029). No significant association was identified between sex and short-term outcome (chi-square test, *p* = 1.0).

### 3.4. Long-Term Follow-Up

Among the 59 foals discharged alive, long-term follow-up information regarding the occurrence of colic episodes after discharge was available for 47 foals (79.7%). Post-discharge colic episodes were reported in 3/47 foals (6.4%). In two foals, colic episodes were transient and resolved with conservative management. In one foal (2.1%), a severe colic episode occurring approximately three months after discharge was correlated to intra-abdominal adhesions and resulted in intraoperative euthanasia. No additional foals were reported to suffer from recurrent colic or to require surgical intervention during the follow-up period. Information regarding the beginning of the intended athletic activity was available only for 36/59 patients (61%). Of these, 28 animals (77.8%) successfully started the expected athletic activity for their breed and intended use. The remaining 8 animals (22.2%) did not start athletic activity; when reported, the reasons were unrelated to gastrointestinal disease and included orthopedic, developmental, or management-related factors. Missing long-term data reflected unavailable owner follow-up and, in the case of athletic outcome, horses not yet at an age suitable for assessment.

**Figure 2:**
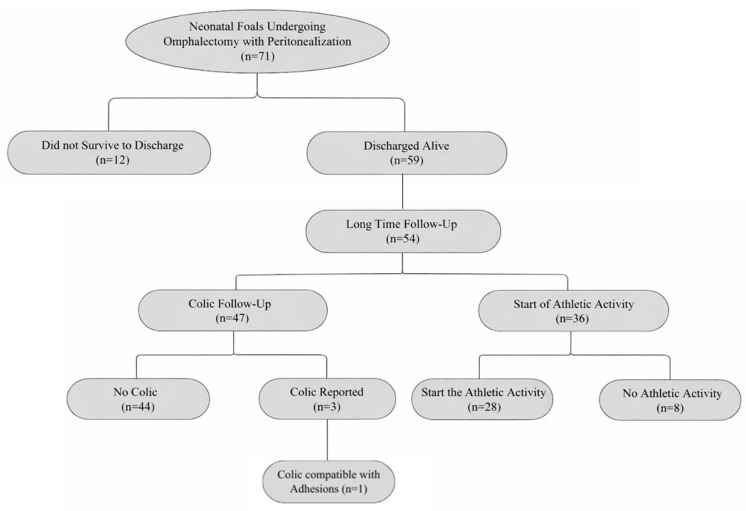
Flow diagram summarizing the study population and short- and long-term outcomes of neonatal foals undergoing omphalectomy with peritonealization of the umbilical arterial stumps.

A summary of the study population, short-term outcome, and availability of long-term follow-up data is provided in Figure 2. Full descriptive statistics and results of inferential analyses are reported in Supplementary Tables S1 and S2.

## 4. Discussion

The main objective of the present study was to evaluate the short- and long-term outcomes of neonatal foals undergoing omphalectomy with peritonealization of the umbilical arterial stumps. The results indicate that this surgical technique was associated with a high survival rate to hospital discharge and a low incidence of clinically relevant long-term abdominal complications, including colic possibly associated with postoperative adhesions.

Within the study population, 12 foals did not survive to hospital discharge, corresponding to a short-term mortality rate of 16.9%. All foals that did not survive to discharge presented with one or more concurrent systemic or orthopedic comorbidities at admission. Foals that did not survive to discharge had a significantly shorter duration of hospitalization compared with survivors, reflecting the severity of their clinical condition and early clinical deterioration. In contrast, age at admission did not differ significantly between survivors and non-survivors. These findings support the interpretation that short-term mortality was primarily driven by systemic disease severity rather than by surgical timing or demographic factors. Previous studies highlighted that Failure of Passive Transfer (FPT) is significantly associated with an increased risk of postoperative complications, as foals with low immunoglobulin levels are more prone to bacteremia and subsequent septic disease [18,19]. Given that sepsis is one of the most common causes of morbidity and mortality in neonatal foals, it is unsurprising that septic states were documented in 4/12 of the foals (33.3%) that died post-operatively, a proportion in agreement with other literature reports [20,21,22].

Long-term follow-up information was available for a subset of foals discharged alive. Post-discharge colic history could be obtained for 47 of the 59 foals (79.7%) that survived to hospital discharge. Among these, 3 foals (6.4%) experienced at least one episode of colic after discharge. Only one foal (1/47; 2.1%) developed a severe colic episode that was considered clinically compatible with postoperative adhesions and resulted in death approximately three months after discharge. When considered relative to the entire study population, this corresponds to an incidence of colic compatible with postoperative adhesions of 1.4% (1/71). No other foals were reported to experience recurrent colic episodes or to require surgical intervention during the follow-up period. The beginning of the intended athletic activity could be assessed in 36 of the 59 foals (61%) that survived to hospital discharge. Among these, 28 foals (77.8%) successfully initiated the expected athletic activity for their breed and intended use, whereas 8 foals (22.2%) did not. When reported, failure to begin the athletic activity was not related to gastrointestinal disease or abdominal complications, but rather to orthopedic, developmental, or management-related factors. For the remaining foals, assessment of athletic outcome was not available primarily because they were still too young at the time of follow-up to have commenced training or athletic activity, particularly in more recent cases.

Consistent with numerous previous reports, the population examined in the present study showed a clear predominance of male foals, which accounted for 70.4% of all cases (50/71) [4,7,23,24,25,26,27]. Male foals appear predisposed to umbilical and urachal disorders because delayed penile–preputial separation may lead to urine retention and periumbilical dermatitis, while a higher incidence of stranguria and meconium retention results in tenesmus and increased intra-abdominal pressure, thereby predisposing to persistence of the urachus [28,29,30]. Furthermore, the longer and narrower urethra in male foals may contribute to increased bladder rupture during parturition [31].

Umbilical arteries, alone or in combination with other umbilical structures, are often involved in the infectious process leading to umbilical disorders [6]. The surgical approach adopted and described in this study includes wrapping the umbilical artery stumps in a peritoneal flap to protect the abdomen from possible contamination and reduce adhesion formation. The peritoneum functions as an active biological drain, supported by an extensive vascular and lymphatic system that facilitates fluid absorption and contributes to the control of infection [32]. The omentalization technique using the omentum—a peritoneal fold—as protection from peritonitis and a healing promoter has been applied for surgical management of pancreatic, prostatic and uterine abscesses in dogs [33,34,35]. Surgical treatment may reduce disease duration, limit the spread of infection, and minimize the need for prolonged antibiotic administration. The low incidence of clinically relevant long-term abdominal complications observed in this study supports the hypothesis that the coverage with visceral peritoneum of the umbilical arterial stumps may contribute to reducing postoperative morbidity, particularly in a population at high risk for inflammatory and septic conditions.

In horses undergoing laparotomy, clinically significant postoperative adhesions are reported in 9% to 33% of cases [13,36,37,38,39]. Adhesions occur more commonly following small-intestine surgery (56%) than large-intestine procedures (44%). Small-intestinal adhesions are also more likely to be clinically significant, with 94% producing symptoms compared to 71% of large-intestinal adhesions. Notably, even in horses operated for large-bowel disease, adhesions tend to develop more frequently in the small intestine [13]. The small intestine is anatomically located near the bladder apex and umbilical artery stumps; for this reason, sealing off the diseased area from surrounding tissue and ensuring direct access of omental pro-healing factors can result in an improvement to the classical remnants resection technique, with the aim of reducing the likelihood of adhesion formation. To the present day, prophylaxis—through meticulous surgical technique combined with anti-inflammatory, antimicrobial, and anticoagulant (heparin) therapy—represents the most effective approach currently available to reduce the risk of abdominal adhesions [15]. Cable et al. reported a 33% incidence of intra-abdominal adhesions in foals undergoing abdominal surgery, mainly for colic, with diagnosis based on a second laparotomy or necropsy, and adhesions being causative in approximately half of the cases [14]. In contrast, the present study used post-discharge colic episodes as a clinically relevant surrogate outcome, as diagnostic imaging has limited accuracy for adhesion detection [40], and a definitive diagnosis would require invasive procedures that are not ethically justifiable in clinically healthy animals. Consequently, subclinical adhesions may have gone undetected, and the true incidence of adhesion formation may be underestimated; however, the aim of this study was to assess clinically relevant long-term morbidity rather than the anatomical prevalence of adhesions.

Overall, timely surgical intervention, appropriate technique, and careful postoperative management proved effective in ensuring rapid recovery and the absence of significant long-term complications [6]. The surgical approach reduces the healing time and the risk of septic hematogenous spread [41]; additionally, another considerable advantage of surgery lies in its potential to limit pharmacological therapy, particularly antibiotic use, to the perioperative and early postoperative periods [42]. Medical management alone traditionally requires prolonged antibiotic administration—2–3 weeks or more—[5], while reduced antibiotic use offers several benefits, including decreased environmental dispersion and mitigation of adverse effects on the patient’s intestinal microbiota, which is especially relevant in debilitated neonates. Although surgery may initially appear more costly, the prolonged hospitalization and extended pharmacological treatment required for medical management may ultimately result in similar or even higher expenses.

The retrospective nature of this study represents an inherent limitation, as data collection depended on the accuracy and completeness of medical records. In addition, long-term outcome assessment was not available for all foals discharged alive. Follow-up regarding post-discharge colic and athletic outcome could be obtained only for a subset of cases, reflecting mainly the young age of several horses that had not yet commenced training at the time of evaluation. Furthermore, the lack of a true control group of foals managed without peritonealization represents an additional limitation; consequently, interpretation of the potential benefits of this technique relies on comparison with previously published data rather than on direct intra-study comparison. The inclusion of a contemporaneous control group of animals would be necessary in future studies to more robustly evaluate the impact of this surgical modification. Moreover, it should be acknowledged that the surgical procedure was limited to the umbilical arterial remnants; therefore, complications arising from concurrent pathology of other umbilical structures cannot be excluded and may have contributed to postoperative or long-term complications. Long-term outcomes were primarily assessed through owner interviews rather than standardized clinical re-examination, and no advanced diagnostic imaging or surgical confirmation was available to definitively identify postoperative adhesions. As a result, long-term findings were interpreted descriptively, and no inferential statistical analysis was undertaken for these outcomes. Finally, the absence of a comparison group of foals treated with alternative surgical techniques limits the ability to directly assess the effect of peritonealization of the umbilical arterial stumps. Future prospective studies with standardized follow-up protocols would be necessary to further clarify the role of this surgical modification in reducing long-term abdominal morbidity.

## 5. Conclusions

In conclusion, omphalectomy with peritonealization of the umbilical arterial stumps was associated with favorable short- and long-term outcomes in the majority of neonatal foals included in this retrospective study. Short-term survival to hospital discharge was high, and short-term mortality was primarily associated with the presence of concurrent systemic or orthopedic comorbidities rather than with age, sex, or surgical timing. Long-term follow-up, although available only for a subset of discharged foals, revealed a low incidence of post-discharge colic and a very low incidence of colic episodes considered possibly associated with postoperative adhesions. In addition, most foals for which athletic outcome could be assessed successfully started their intended activity, with failure to do so mainly related to non-gastrointestinal factors or to young age at the time of follow-up. Although direct comparison with traditional omphalectomy techniques was not possible in the present study, the low rate of clinically relevant long-term abdominal complications observed in this cohort is consistent with a favorable long-term outcome following this surgical approach. Taken together, these findings suggest that peritonealization of the umbilical arterial stumps may represent a safe surgical option in neonatal foals undergoing omphalectomy. Further prospective, controlled studies are warranted to confirm these observations and to directly compare peritonealization with conventional surgical approaches, including potential application of the technique to the umbilical vein stump.

## Data Availability

The data supporting the findings of this study are not publicly available due to privacy and institutional restrictions but may be provided by the corresponding author upon reasonable request.

## Supplementary Materials

The following supporting information can be downloaded at: https://www.mdpi.com/article/10.3390/ani16040551/s1, Table S1: Descriptive statistics of the study population (n = 71), Table S2: Summary of inferential statistical analyses for associations with short-term outcome.
