# Supplementary material for: Short- and Long-Term Outcome of 71 Foals Undergoing Omphalectomy with Peritonealization of Arterial Stumps
Source: Animals (Basel). 2026 Feb 10;16(4):551. doi: 10.3390/ani16040551 (PMC12937459; doi:10.3390/ani16040551)
Supplement: Supplementary file 1 [file animals-16-00551-s001.zip › animals-4101289-supplementary.pdf]

Supplementary Matherials

Variable	n	Mean ± SD	Median (IQR)	Min–Max
Age at admission (days)	71	6.6 ± 5.1	5 (3–9)	1–27
Length of hospitalization (days)	71	15.2 ± 9.8	13 (9–20)	2–49

Supplementary Table S1. Descriptive statistics of the study population (n = 71)

Variable	Comparison	Test	p-value
Age at admission	Survived vs not survived	Mann–Whitney U	0.233
Length of hospitalization	Survived vs not survived	Mann–Whitney U	**0.011**
≥1 concurrent comorbidity	Survived vs not survived	Fisher's exact	**0.029**
Postoperative complications	Survived vs not survived	Fisher's exact	**0.039**
Sex	Survived vs not survived	Chi-square	1.000

Supplementary Table S2. Summary of inferential statistical analyses for associations with short-term outcome
